# Preclinical Immune Response and Safety Evaluation of the Protein Subunit Vaccine Nanocovax for COVID-19

**DOI:** 10.3389/fimmu.2021.766112

**Published:** 2021-12-06

**Authors:** Thi Nhu Mai Tran, Bruce Pearson May, Trong Thuan Ung, Mai Khoi Nguyen, Thi Thuy Trang Nguyen, Van Long Dinh, Chinh Chung Doan, The Vinh Tran, Hiep Khong, Thi Thanh Truc Nguyen, Hoang Quoc Huy Hua, Viet Anh Nguyen, Tan Phat Ha, Dang Luu Phan, Truong An Nguyen, Thi Ngoc Bui, Tieu My Tu, Thi Theo Nguyen, Thi Thuy Hang Le, Thi Lan Dong, Trong Hieu Huynh, Phien Huong Ho, Nguyen Thanh Thao Le, Cong Thao Truong, Hoang Phi Pham, Cong Y. Luong, Nie Lim Y, Minh Ngoc Cao, Duy Khanh Nguyen, Thi Thanh Le, Duc Cuong Vuong, Le Khanh Hang Nguyen, Minh Si Do

**Affiliations:** ^1^ Department of Research and Development, Nanogen Pharmaceutical Biotechnology Joint Stock Company (JSC), Ho Chi Minh City, Vietnam; ^2^ Department of Virology, National Institute of Hygiene and Epidemiology (NIHE), Hanoi, Vietnam

**Keywords:** COVID-19, SARS-CoV-2, RBD, ACE2, CHO

## Abstract

The coronavirus disease 2019 (COVID-19) pandemic caused by the severe acute respiratory syndrome coronavirus 2 (SARS-CoV-2) has become a global health concern. The development of vaccines with high immunogenicity and safety is crucial for controlling the global COVID-19 pandemic and preventing further illness and fatalities. Here, we report the development of a SARS-CoV-2 vaccine candidate, Nanocovax, based on recombinant protein production of the extracellular (soluble) portion of the spike (S) protein of SARS-CoV-2. The results showed that Nanocovax induced high levels of S protein-specific IgG and neutralizing antibodies in three animal models: BALB/c mouse, Syrian hamster, and a non-human primate (*Macaca leonina*). In addition, a viral challenge study using the hamster model showed that Nanocovax protected the upper respiratory tract from SARS-CoV-2 infection. Nanocovax did not induce any adverse effects in mice (*Mus musculus* var. albino) and rats (*Rattus norvegicus*). These preclinical results indicate that Nanocovax is safe and effective.

## 1 Introduction

The coronavirus disease 2019 (COVID-19) pandemic caused by the severe acute respiratory syndrome coronavirus 2 (SARS-CoV-2) has become a global health emergency (1). Since it was first reported in Wuhan, China, at the end of 2019, there have been over 165 million cases worldwide and nearly 3.5 million deaths as of May 2021 (2), with no obvious short-term resolution. Like SARS-CoV (79% genomic sequence identity) (3), SARS-CoV-2 utilizes the receptor angiotensin-converting enzyme 2 (ACE2), which is expressed on numerous cells (including lung, heart, kidney, and intestine) as the entry fusion receptor by its viral spike, a homotrimeric complex of spike (S) proteins (4). The S protein is a homomeric class I fusion protein with each S monomer containing the N-terminal S1 subunit, which includes the receptor-binding domain (RBD) and the C-terminal S2 subunit, which is anchored to the viral membrane and is required for trimerization of the spike itself and fusion to the host membrane (5). During fusion to host cell membranes, the S protein undergoes extensive conformational changes that cause dissociation of the S1 subunit from the complex and the formation of a stable post-fusion conformation of the S2 subunit (6). Therefore, the S protein of SARS-CoV-2 plays a vital role in the invasive process.

Potential vaccines against SARS-CoV-2 have focused on the S protein and include mRNA-lipid nanoparticles that encode the S protein, viral vectored DNA-based vaccines (notably recombinant adenoviruses), and subunit vaccines that contain purified S protein (7, 8). The WHO has estimated that 102 and 185 S protein-targeted vaccines are in the clinical and preclinical development phases, respectively (10). The vaccine candidates that have been developed or are under development include recombinant protein vaccines, inactivated vaccines, viral vector-based vaccines, and DNA vaccines to prevent virus infection. Currently, three vaccines are approved by the Food and Drug Administration (FDA): BNT162b2 is a lipid nanoparticle-formulated, nucleoside-modified mRNA vaccine (Comirnaty^®^; Pfizer/BionNtech) (11); the Moderna COVID-19 vaccine is mRNA-based (12); and Janssen (Johnson and Johnson) (13) is based on an adenovirus vector and is used in adults aged 18 years and older. Besides vaccines, oligonucleotides, peptides, interferon, and small-molecule drugs have been suggested to control SARS-CoV-2 infection (14). According to the FDA during a public health emergency, three anti-SARS-CoV-2 monoclonal antibody (mAb) therapies have been approved for the treatment of non-hospitalized patients with mild-to-moderate COVID-19, including bamlanivimab, etesevimab, or casirivimab with imdevimab (15).

In this study, we have developed a COVID-19 subunit vaccine, named Nanocovax, based on recombinant protein technology to produce the extracellular (soluble) portion of the S protein of SARS-CoV-2. In brief, a gene encoding the S protein was constructed using the wild-type sequence of the S protein extracellular domain. The construct was transfected into Chinese hamster ovary (CHO) cells—the product was named CHO-spike cells—with the highest S protein expression selected. The S protein of SARS-CoV-2 was then absorbed into aluminum hydroxide gel adjuvant (Alhydrogel^®^; Croda, Denmark). Here, we describe the preclinical studies of the Nanocovax vaccine and illustrate its immunogenicity, efficacy, and safety in mouse, hamster, non-human primate, and rat models.

## 2 Materials and Methods

### 2.1 Plasmid Construction, Cell Clone, and Purification

#### 2.1.1 Plasmid Construction

The pCNeoMEM vector was used as the expression vector. The pCNeoMEM plasmid contained a G418 resistance gene used as a selection marker, a MoMLV promoter to express the target gene, a human ubiquitous chromatin opening element (UCOE), untranslated regions from the Chinese hamster EEF1A1 gene (eEF1A1), and a synthetic matrix attachment sequence (sMAR). The gene encoding the spike protein of SARS-CoV-2 (UniProt P0DTC2), codon-optimized for expression in CHO cells, was synthesized by GenScript (Piscataway, NJ, USA). The optimized DNA fragment was cloned into the expression vector to create pCNeoMEM-S, which was completely sequenced using NextGen Technology at the Center for Computational and Integrative Biology, Harvard University.

#### 2.1.2 Cell Culture and Protein Expression

CHO cells (cGMP bank, Thermo Fisher Scientific, Waltham, MA, USA) were propagated and maintained in the animal component-free, chemically defined medium, PowerCHO-2 (Lonza, Walkersville, MD, USA) at 37°C and 5% CO_2_. Suspension cells were routinely subcultured every 2–3 days at a cell concentration of 2 × 10^5^ cells/ml. Before transfection, the cells were seeded at 1 × 10^6^ cells/ml in a 6-well plate and cultured for 24 h. For transfection, Lipofectamine LTX Reagent (Thermo Fisher Scientific) was used following the manufacturer’s instructions. Under optimized conditions, 3 µg of plasmids was transfected into 1 × 10^6^ cells per well in 6-well plates using PowerCHO-2 medium. After 48 h of transfection, the expression of the S protein was evaluated using the ELISA. After transient transfection, selection was performed by culturing the transfected cells in PowerCHO-2 medium supplemented with 400 µg/ml of Geneticin (G418; Sigma-Aldrich, St. Louis, MO, USA). The selective medium was replaced every 2 to 3 days for 3 weeks to obtain stable cell lines. Single-cell cloning was initiated by limiting dilution. The clones with higher productivity were selected to create a master cell bank and a working cell bank. Process development was performed using the high-throughput multi-parallel bioreactors Ambr 15 and Ambr 250 (Sartorius Stedim Biotech, Göttingen, Germany). Protein production at the pilot scale for non-clinical trial material was archived in a 500-L bioreactor (BIOSTAT STR^®^ 500; Sartorius Stedim Biotech, Göttingen, Germany) using a fed-batch process with PowerCHO-2 as the basal medium, supplemented with Feed 3 (ExcellGene SA, Monthey, Switzerland), trace elements, and activated sugars (n = 3).

#### 2.1.3 Protein Purification

S protein was purified from the cell broth using an ÄKTA Pilot 600R system (GE Healthcare, Little Chalfont, UK). After harvest, the cell supernatant was clarified by depth-filtration (Merck Millipore, Darmstadt, Germany) to remove cells and purified using consecutive chromatography steps. First, the sample was loaded onto a Blue Sepharose column (Cytiva, Marlborough, MA, USA) to specifically collect S proteins and then treated at a low pH (3.2–3.5) for 60–70 min to inactivate the virus. Next, the sample was passed through a Q membrane, and then a phenyl membrane (Sartorius Stedim Biotech, Göttingen, Germany), to remove host cell DNA, proteins, and endotoxins. Exotic viruses were removed by nano-filtration. Purified S proteins were exchanged with storage buffer and filtered through a 0.22-μm filter (Sartorius Stedim Biotech, Göttingen, Germany). The concentration of the purified protein was determined using ELISA. The purity of the protein was determined using size-exclusion high-performance liquid chromatography (SE-HPLC), residual host cell DNA assay, and residual host cell protein assay.

### 2.2 SARS-CoV-2 Spike Protein Analysis

#### 2.2.1 Spike Protein Identification

##### 2.2.1.1 Sodium Dodecyl Sulfate–Polyacrylamide Gel Electrophoresis and Western Blotting Assay

Purified proteins were mixed with loading buffer and loaded onto sodium dodecyl sulfate–polyacrylamide gel electrophoresis (SDS-PAGE) gels. Proteins were electrophoresed for 110 min at 80 V and 500 mA and visualized using Coomassie Brilliant Blue G250 staining (Sigma-Aldrich) or transferred to a nitrocellulose blotting membrane (Sigma-Aldrich) at a constant current of 250 mA for 90 min. Next, the membranes were blocked in 0.5% bovine serum albumin (BSA) (Sigma-Aldrich) and incubated with human anti-S1 antibody (Abcam, Cambridge, UK) for 3 h at room temperature. After being washed, the membranes were incubated with horseradish peroxidase (HRP)-linked rabbit anti-human IgG antibody (Abcam) for 1 h at room temperature. Membranes were washed three times with PBST [1× phosphate-buffered saline (PBS), 0.1% Tween^®^ 20 Detergent], and the protein bands were visualized by enhancing the TMB (3,3′, 5,5′-tetramethylbenzidine) substrate solution (Sigma-Aldrich).

##### 2.2.1.2 Enzyme-Linked Immunosorbent Assay

The culture supernatant sample or purified S protein was coated onto each well of the microtiter plate and left overnight at 4°C. After the wells were blocked with 1% BSA for 1 h, dilutions of human anti-S1 antibody (Abcam) were added to the wells and incubated for 3 h at room temperature. After being washed, HRP-conjugated rabbit anti-human IgG antibody (Abcam) was added and incubated for 1 h at room temperature. The bound antibodies were detected using TMB substrate (Sigma-Aldrich). Absorbance was read at 450 nm using a Multimode Plate Reader (Promega, Madison, WI, USA).

#### 2.2.2 Structure Analysis

##### 2.2.2.1 Molecular Mass Assay

The molecular mass of the purified protein was measured by Biofidus AG Analytical Services (Bielefeld, Germany) using matrix-assisted laser desorption/ionization–time-of-flight mass spectrometry (MALDI-TOF/MS). In brief, a recombinant SARS-CoV-2 S protein was desalted and concentrated with C4 ZipTips (Merck Millipore) and spotted onto a ground steel target using 2′,5′-dihydroxyacetophenone. The sample was measured using MALDI-TOF/MS (ultrafleXtreme; Bruker Daltonik GmbH, Bremen, Germany) in positive ion mode. The recorded MS spectra were processed using FlexAnalysis software (Bruker Daltonik GmbH).

##### 2.2.2.2 Peptide Mapping Assay

Peptide mapping of the purified protein was performed by Biofidus AG Analytical Services. Briefly, peptide mapping of a recombinant SARS-CoV-2 S protein was measured by LC-electrospray ionization–MS (LC-ESI-MS) using different digestion strategies. The focus of peptide mapping was sequence verification and analysis of N- and C-terminal modifications. MS analysis was performed using a compact quadrupole TOF (Q-TOF) mass spectrometer (Bruker Daltonik GmbH). The recorded LC-ESI-MS and tandem MS (MS/MS) spectra were processed, annotated, and searched against a customized sequence database using the Mascot search engine (Matrix Science, London, UK). Modified peptides were identified by their exact mass and retention time and quantified by their mass spectrometric signal intensity.

### 2.3 *In Vivo* Immunogenicity Evaluation of Nanocovax Vaccine in BALB/c Mouse, Syrian Hamster, and Non-Human Primate Models

#### 2.3.1 Animal Vaccination

BALB/c mice (6–10 weeks old), Syrian hamsters (*Mesocricetus auratus*: 8–12 weeks old), and northern pig-tailed macaques (*Macaca leonina*: 4–5 years old) were used for immunological studies. They were immunized intramuscularly (IM) with Nanocovax at doses of 25, 50, 75, and 100 µg; and serum samples were collected for the quantification of S protein-specific IgG antibodies by ELISA. Blood samples were collected, allowed to clot at room temperature for 60 min, and then centrifuged at 1,000 × *g* for 15 min. The upper serum fraction was collected and heat-inactivated at 56°C for 30 min before use or kept at –20°C.

#### 2.3.2 *In Vitro* Surrogate Virus Neutralization Assay

The virus neutralization abilities of antibodies in the sera of BALB/c mice, hamsters, and macaques were determined using the Surrogate Virus Neutralization Test (cat# L00847, GenScript, Singapore). The percentages of neutralized virus in the sera were determined according to the manufacturer’s protocol.

#### 2.3.3 *In Vitro* SARS-CoV-2 Virus Neutralization Assay (Plaque Reduction Neutralization Test)

The plaque reduction neutralization test (PRNT) detects and quantifies the neutralizing antibody SARS-CoV-2 in serum samples. Sera were twofold serially diluted in culture medium with a starting dilution of 1:20. The diluted sera were mixed with 100 plaque-forming units (PFU) of the SARS-CoV-2 virus for 1 h at 37°C. The virus–serum mixtures were added to Vero E6 cell monolayers and incubated for 1 h at 37°C in a 5% CO_2_ incubator. The plates were then overlaid with 1% agarose in cell culture medium and incubated for 4 days when the plates were fixed and stained. Antibody titers were defined as the highest serum dilution that resulted in a >50% (PRNT_50_) reduction in the number of plaques. The PRNT was performed in duplicate using 24-well tissue culture plates in a biosafety level 3 facility at the National Institute of Hygiene and Epidemiology, Hanoi, Vietnam, adapted from Okba et al. (16).

### 2.4 Protective Efficacy Evaluation of Nanocovax Vaccine in Syrian Hamsters

#### 2.4.1 Viral Challenge Study

The hamsters were assigned to the following groups: 1) vaccinated with Nanocovax on days 0 and 7 and then challenged with a high level of the SARS-CoV-2 virus on day 14 by the intranasal route (TCID_50_ = 2 × 10^5^); 2) vaccinated with Nanocovax on days 0 and 7 and then challenged with a low level of the SARS-CoV-2 virus on day 14 by the intranasal route (TCID_50_ = 1 × 10^3^); and 3) injected with placebo (PBS) and challenged with a high/low level of the SARS-CoV-2 virus on day 14 by the intranasal route (TCID_50_ = 2 × 10^5^ and 1 × 10^3^). The baseline body weights were measured before infection. Animals were monitored for signs of morbidity (such as weight loss, ruffled hair, and sweating) for 14 days. On day 28, the lungs were collected for SARS-CoV-2 detection by real-time RT-PCR. The method for the quantitative detection of SARS-CoV-2 in lung samples was adapted from the WHO protocol (17). Infection doses were chosen based on the study by Imai et al. (18).

#### 2.4.2 Real-Time RT-PCR

In this study, real-time RT-PCR was performed to quantify the SARS-CoV-2 level. This PCR amplified the *envelope* (E) gene of SARS-CoV-2 using the forward primer 5′-ACAGGTACGTTAATAGTTAATAGC-3′; reverse primer: 5′-ATATTGCAGCAGTACGCA-CAC-3′; and probe: 5′-FAMACACTAGCCATCCTTACTGCGCTTCGBBQ-3′. Real-time RT-PCR assays were conducted using a TaqMan One-Step RT-PCR kit (Thermo Fisher Scientific) on a Real-Time PCR System (Bio-Rad, Hercules, CA, USA) with the following cycling conditions: 55°C for 10 min for reverse transcription, 95°C for 3 min, and 45 cycles of 95°C for 15 s and 58°C for 30 s. The absolute copy number of viral loads was determined using serially diluted DNA control targeting the E gene of SARS-CoV-2.

### 2.5 Safety Evaluation of Nanocovax Vaccine

According to the International Council for Harmonisation of Technical Requirements for Pharmaceuticals for Human Use (ICH)/Good Laboratory Practices (GLP) guidelines with minor modifications, single-dose and repeat-dose toxicity studies were performed on adult male and female mice and rats, with a few modifications. The animals were carefully examined and weighed before the start of the experiment. In the single-dose toxicity test, 60 mice of both sexes were divided into six groups (n = 10, five females and five males) and IM injected with Nanocovax at single doses of 25, 50, 75, and 100 µg, or with the placebo. Untreated mice were used as biological controls. All animals were regularly monitored continuously within the first 4 h for behavioral and pathological signs and then daily for the next 14 days for mortality, abnormal behavior, and body weight. In the repeat-dose toxicity test, a total of 36 rats were divided into six groups (n = 6; three males and three females). Rats were IM injected with Nanocovax at daily doses of 25, 50, 75, and 100 µg, or the placebo, for 28 days. Untreated rats were used as biological controls. Mortality and clinical signs were observed daily, and body weight was determined at the indicated time points during the experimental period. At the end of treatment, all tested rats were anesthetized to collect blood samples for the analysis of biochemical and hematological parameters. Following the sacrifice of the animals, three vital organs (kidneys, spleen, and liver) were immediately isolated, weighed individually, and examined histologically.

### 2.6 Animal Ethics Statement

This study was carried out in strict adherence to the guidelines of the Animal Laboratory of Nanogen Pharmaceutical Company, the National Institute of Drug Quality Control (NIDQC), and the laboratory of Hanoi Medical University (HMU). The processes were designed according to the guidelines of the ICH–Good Clinical Practice (GCP), Drug Administration of Vietnam, and Association of Southeast Asian Nations (ASEAN) Common Technical Dossier (ACTD) and approved by the Ethics Committee of the Ministry of Health.

### 2.7 Statistical Analysis

The collected data were statistically analyzed using GraphPad Prism, version 5 (GraphPad Software Inc., San Diego, CA, USA). Data are expressed as the mean ± SD. Statistical analysis was performed using two-way ANOVA with Bonferroni *post-hoc* tests and one-way ANOVA followed by the Newman–Keuls multiple comparison test to assess the differences between the various groups. Differences described as significant in the text correspond to *p < 0.05, **p < 0.01, and ***p < 0.001.

## 3 Results

### 3.1 Recombinant SAR-CoV-2 S Protein Production

**Figure 1:**
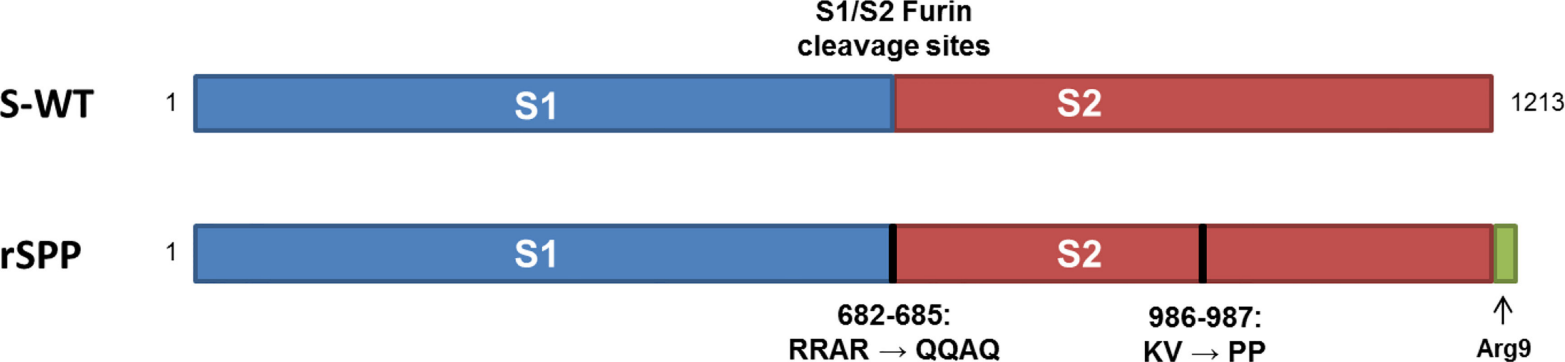
Recombinant SARS-CoV-2 spike (S) protein construct used for Nanocovax. Recombinant S protein (rSPP) construct containing native S protein sequence (aa 1–1213) followed by the arginine-9 tag. The S1/S2 furin cleavage sites (RRAR) and two amino acids (Kv) were mutated as noted.

To generate the SAR-CoV-2 antigen for vaccine development, we designed an optimized DNA sequence encoding the extracellular domain sequence of the S protein, which has some changes in 1) the S1/S2 furin cleavage site to minimize the cleavage of S1/S2 during protein production, 2) the two proline residues in the S2 domain (986-987) to enhance prefusion-stabilized SARS-CoV-2 spikes, and 3) the 9-arginine residue in the C-terminus (**Figure 1**). The recombinant SAR-CoV-2 S protein-encoding clone was selected on the basis of phenotypic stability, productivity, and key quality properties of the desired product, named CHO-spike cells. The nucleotide sequence of the gene integrated into the CHO-spike cell genome was confirmed by sequencing complementary DNA (data not shown).

**Figure 2:**
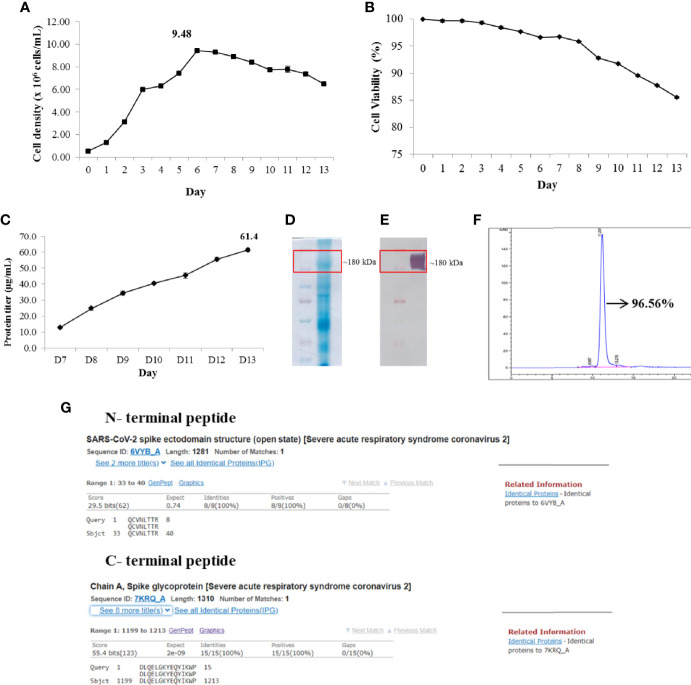
High-yield production and characterization of recombinant SARS-CoV-2 spike (S) protein. Viable cell density **(A)**. Viability of cells **(B)**. Protein titer from day 7 to end of fed-batch culture **(C)**. Sodium dodecyl sulfate–polyacrylamide gel electrophoresis (SDS-PAGE) of harvest sample **(D)**. Western blotting of harvest sample **(E)**. Purity of S protein **(F)**. N- and C-terminal peptide sequencing and blasting to complete non-redundant database of protein sequence storage at the National Center for Biotechnology Information (NCBI) **(G)**.

Upstream production of S proteins by CHO-spike cells was performed in a 500-L bioreactor using a fed-batch process with Feed 3 supplement. The cells were cultured in 380 L of PowerCHO-2 medium at an initial concentration of 0.5 × 10^6^ cells/ml and fed with 1.5% of the final volume of Feed 3 daily from day 3 to day 13. The temperature was initially 37°C and was changed to 32°C on day 3 of the cell culture. Cell density, cell viability, and protein titer were determined at the indicated time points. **Figure 2** shows the high performance of CHO-spike cells at a scale of 500 L using the optimized fed-batch protocol. The CHO-spike cells obtained a maximum cell density of 9.48 ± 0.13 × 10^6^ cells/ml (**Figure 2A**) and survived for 13 days with a cell viability of 85% (**Figure 2B**). At the end of cultivation, the culture supernatant was harvested and purified for further analysis. The data showed that the maximum protein titer was 61.4 ± 1.06 mg/L (**Figure 2C**) on the day of harvest. SDS-PAGE of the harvested sample indicated that the predicted protein was approximately 180 kDa, similar to the molecular weight of the S protein (**Figure 2D**). In addition, this protein bound specifically to the anti-S1 protein antibody (**Figure 2E**). The harvested sample was purified using an ÄKTA Pilot 600R system. The results of the ELISA assay also indicated that the purified protein concentration was 21.4 ± 0.18 g per batch with 96.56% purity (**Figure 2F**). In addition, residual host cell proteins and DNA were not detected in the purified S protein (data not shown).

### 3.2 Characterization of Recombinant SARS-CoV-2 S Protein

The intact mass analysis data by MALDI-MS also confirmed that our S protein mass was 185,668 ± 1,849 Da (**Supplementary Data S1**). In contrast, the N- and C-terminal sequence and peptide mapping of recombinant S protein suggested truncation of N-terminal serine and complete pyroglutamate formation of the N-terminal glutamine. In addition, the C-terminus showed high heterogeneity with a C-terminal peptide, with truncation of aa 1215–1222 being the most abundant variant (**Supplementary Data S2**). Furthermore, the conservation of recombinant S protein was evaluated by comparing the N- and C-terminal peptide sequences with a complete non-redundant database of protein sequences stored at the National Center for Biotechnology Information (NCBI), using the BLASTP computer program. The analysis data showed that the N- and C-terminal peptide sequences matched 100% to published SARS-CoV-2 spike protein sequences (**Figure 2G**).

### 3.3 Immunogenicity of Nanocovax Vaccine in Animal Models

**Figure 3:**
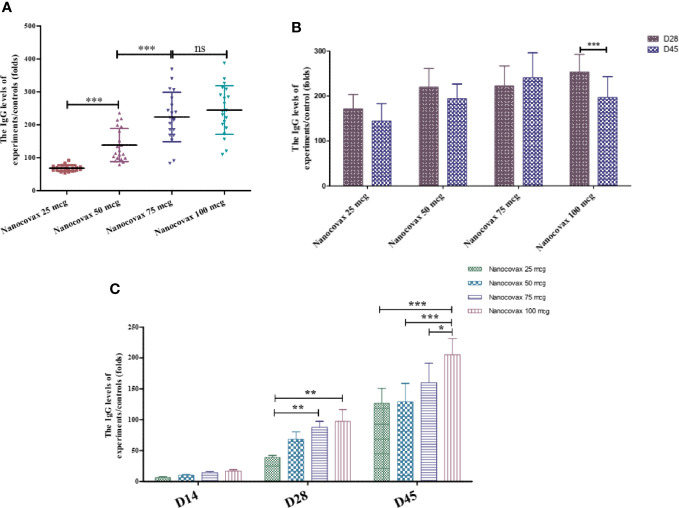
Immunogenicity of Nanocovax vaccine using different animal models. The BALB/c mice were intramuscularly injected twice 7 days apart. Blood was collected on day 14 to determine SARS-CoV-2 S protein-specific antibody IgG levels by ELISA **(A)**. Syrian hamsters were intramuscularly injected twice 7 days apart. Blood was collected on day 28 and day 45 to determine SARS-CoV-2 S protein-specific antibody IgG levels by ELISA assay **(B)**. Northern pig-tailed macaques were intramuscularly injected twice 7 days apart. Blood was collected on days 14, 28, and 45 to determine SARS-CoV-2 S protein-specific antibody IgG levels by ELISA assay **(C)**. The data represent the mean ± SD, and *p*-values were determined by one-way ANOVA with the Newman–Keuls multiple comparison test and two-way ANOVA analysis with Bonferroni *post-hoc* tests (*p < 0.05; **p < 0.01; ***p < 0.001; ns, not significant).

To assess the immunogenicity of Nanocovax, BALB/c mice were injected with various dosages (25, 50, 75, and 100 µg) of vaccine absorbed with 0.5 mg of Al^3+^ (aluminum hydroxide adjuvant) twice, with 7 days between injections. The levels of total specific IgG were determined by ELISA on day 14 post priming injection. The results shown in **Figure 3A** indicate that the IgG levels significantly increased in a dose-dependent manner. To further evaluate the immunogenicity of Nanocovax, Syrian hamsters were vaccinated with various doses of Nanocovax (25, 50, 75, and 100 µg). The antibodies were detected on days 28 and 45 post priming. The data from **Figure 3B** show that the amounts of S protein-specific IgG in the 25-, 50-, 75-, and 100-µg-vaccinated hamster groups on day 28 were 171.5-fold, 219.8-fold, 222.6-fold, and 253.0-fold that in the placebo group, respectively. The level of antibodies was slightly decreased on day 45 in the experiments, at 144.0-fold, 193.8-fold, 240.5-fold, and 196.6-fold than in the placebo group, respectively.

To confirm the immunogenicity of the Nanocovax vaccine, northern pig-tailed macaques were studied. Monkeys were administered twice by IM injection with various concentrations of Nanocovax (25, 50, 75, and 100 µg), or with PBS as a negative control. After the booster injection on day 7, blood samples were collected on days 14, 28, and 45 to detect the level of antibodies. The data in **Figure 3C** indicate that the levels of S protein-specific IgG in the vaccinated groups were significantly increased in a time-dependent manner. On day 28 post priming injection, the levels of S protein-specific IgG were slightly increased in all vaccinated groups, at 39.02-fold, 68.58-fold, 87.82-fold, and 97.37-fold than in the control group in the 25-, 50-, 75-, and 100-µg groups, respectively. Similarly, on day 45, the amounts of S protein-specific IgG in the sera in all vaccinated groups were significantly higher than those in the control group (IgG levels of 25-, 50-, 75-, and 100-µg-vaccinated monkeys were 126.5-fold, 129.1-fold, 159.95-fold, and 205.12-fold, respectively).

**Figure 4:**
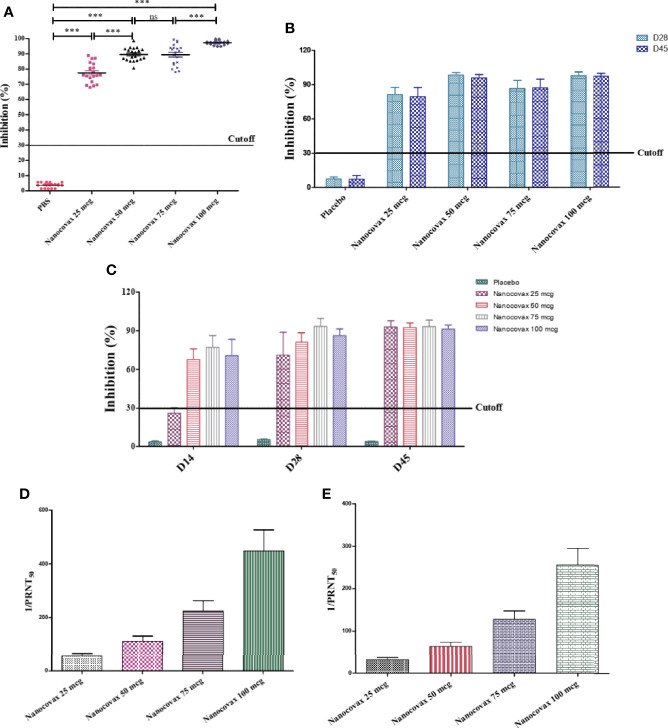
SARS-CoV-2-neutralizing antibodies. BALB/c mice were intramuscularly injected twice 7 days apart. Blood was collected on day 14 to determine SARS-CoV-2-neutralizing antibodies by *in vitro* surrogate virus neutralization assay** (A)**. Syrian hamsters were intramuscularly injected twice 7 days apart. Blood was collected on days 28 and 45 to determine SARS-CoV-2-neutralizing antibodies by *in vitro* surrogate virus neutralization assay **(B)**. Northern pig-tailed macaques were intramuscularly injected twice 7 days apart. Blood was collected on days 14, 28, and 45 to determine SARS-CoV-2-neutralizing antibodies by *in vitro* surrogate virus neutralization assay **(C)**. The SARS-CoV-2 neutralizing antibodies in the BALB/c **(D)** and hamster **(E)** models were also measured by PRNT_50_ assay. The data represent the mean ± SD, and *p*-values were determined by one-way ANOVA with the Newman–Keuls multiple comparison test and two-way ANOVA analysis with Bonferroni *post-hoc* tests (***p < 0.001; ns, not significant).

In contrast, sera were collected from vaccinated animal models, and the percentage of neutralizing antibodies was measured using a surrogate virus neutralization test (SARS-CoV-2 sVNT Kit). For the BALB/c mice model, blood was collected from the mice after immunization on day 14 to detect the percentage of inhibition. As shown in **Figure 4A**, the four dosages of Nanocovax (25, 50, 75, and 100 µg) strongly inhibited SARS-CoV-2 virus compared with the PBS group (***p < 0.001). The inhibition by Nanocovax 50 µg was not significantly different from that by Nanocovax 75 µg (89.60% versus 89.40%). A significant difference was observed between the 25-µg group and the 50-µg group (77.5% versus 89.60%), as well as between the 75-µg and 100-µg groups (89.40% versus 97.30%). For the Syrian hamster model, blood was collected on days 28 and 45 post priming injection with the four dosages of Nanocovax. The percentage inhibition was detected using the SARS-CoV-2 sVNT Kit. As shown in **Figure 4B**, no significant difference (*p* > 0.05) was observed among all groups on day 28 and day 45 post priming injection with four dosages of Nanocovax vaccine (25, 50, 75, and 100 µg). Otherwise, the vaccinated hamster groups showed significant inhibition by antibodies in sera compared with that in the non-vaccinated hamster group (***p < 0.001). Next, we measured the virus neutralization ability of antibodies in a non-human primate model. The monkeys were immunized twice a week with Nanocovax at dosages of 25, 50, 75, and 100 µg. Sera were collected on days 14, 28, and 45 after priming IM injection to determine the inhibition of the S protein using the virus surrogate neutralization kit. The results in **Figure 4C** show that the monkeys vaccinated with the 25-µg dosage of Nanocovax produced no significant inhibition on day 14 (the percent of inhibition was 26.14%, lower than the 30% cutoff value). On days 28 and 45, the monkey groups immunized with the four dosages of Nanocovax had positive results (>30% cutoff value) and showed no significant differences in the percentages of inhibition.

Furthermore, SARS-CoV-2-neutralizing antibodies can be measured by the PRNT_50_ viral neutralization assay, which is used to determine the levels of neutralizing antibodies in the sera of vaccinated animals. The results in **Figure 4D** show that the specimens of BALB/c immunized with Nanocovax had SARS-CoV-2-neutralizing antibodies (1/PRNT_50_) titers of 40–640. Similarly, all specimens of hamsters that received the Nanocovax vaccine had a SARS-CoV-2-neutralizing (1/PRNT_50_) titer of 20–320 (**Figure 4E**).

### 3.4 Protective Efficacy of Nanocovax Vaccine in Hamster Model

**Figure 5:**
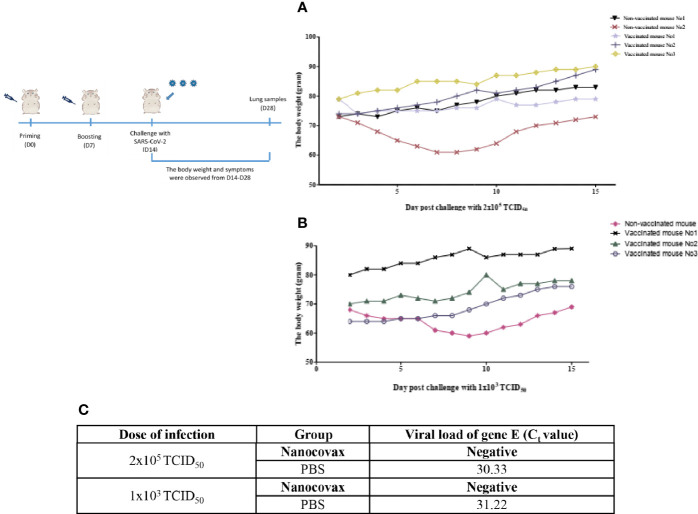
Challenge of Syrian hamsters with live SARS-CoV-2. Syrian hamsters were vaccinated twice 7 days apart. After vaccination, the hamsters were challenged with low and high levels of the SARS-CoV-2 virus on day 14. The weight gain in hamsters was monitored after challenge with high **(A)** and low levels of SARS-CoV-2 **(B)**. SARS-CoV-2 load [*envelope* (E) gene] in lung samples 14 days post challenge was determined by real-time RT-PCR **(C)**.

After challenge with a high or low level of the SARS-CoV-2 virus, three vaccinated hamsters from each group showed no signs of weight loss; they showed weight maintenance for 1 to 2 days post infection, and weight gain continued from day 3 post infection and day 14 post infection (11.8% to 14.5%). No symptoms were observed in vaccinated animals; no shortness of breath, ruffled fur, or lethargy was observed in any vaccinated hamsters that received low and high doses of SARS-CoV-2. Three control hamsters exhibited ruffled fur, lethargy, and sweating symptoms on days 1 and 2 after the virus challenge. Two of the three animals showed severe weight loss by day 7 or 8 post infection (13.2%–16.4%) and gained weight slowly from day 8 or 9 after the challenge test (**Figures 5A, B**).

On day 28, the lungs from the vaccinated and non-vaccinated hamster groups were collected to detect SARS-CoV-2 by real-time RT-PCR. The results in **Figure 5C** show that no SARS-CoV-2 virus-specific RNA was detected in lung samples of the vaccinated group after 14 days of challenge with viral concentrations of 2 × 10^5^ TCID_50_ and 1 × 10^3^ TCID_50_ compared with the non-vaccinated group [cycle threshold (Ct) = 30.33, and Ct = 31.22].

### 3.5 Safety Evaluation of Nanocovax Vaccine

**Figure 6:**
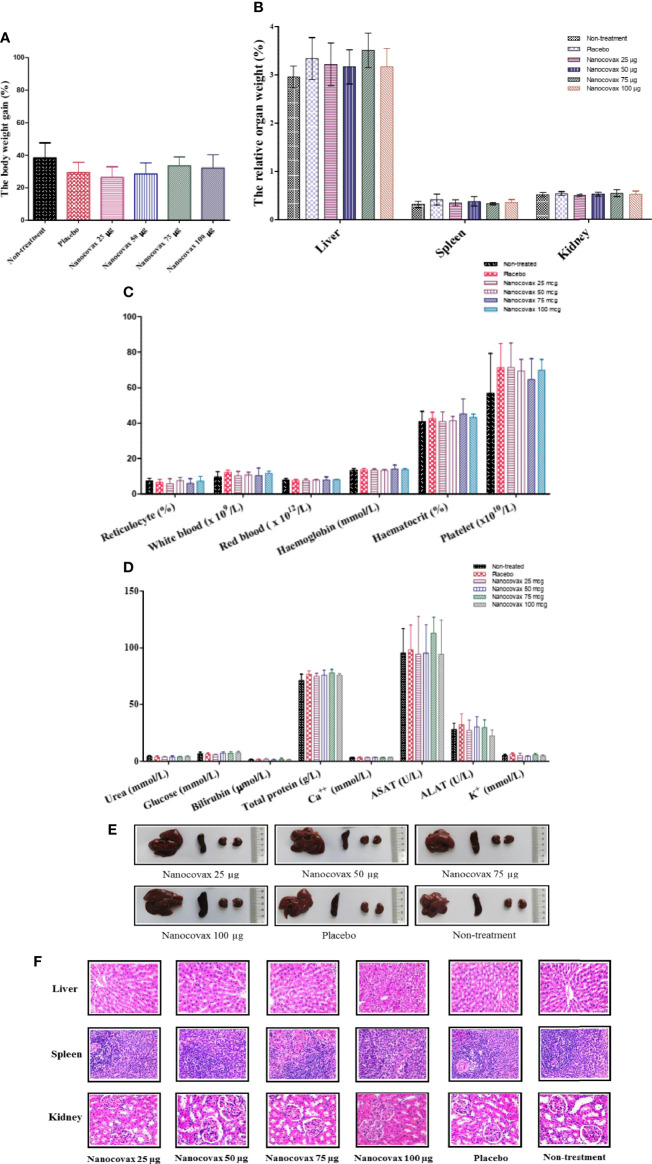
Repeat-dose toxicity study. The repeat-dose toxicity assay was performed on *Rattus norvegicus* by intramuscular injection once a week for 4 weeks. Pathological signals and weight changes were observed. Body weight gain **(A)**. Relative organ weights **(B)**. Indicators of hematological parameters **(C)**. Biochemical parameters **(D)**. Anatomical structure of the organs **(E)** and histological analysis of the liver, spleen, and kidneys **(F)** in repeat-dose test (magnification ×40). The data represent the mean ± SD, and *p*-values were determined by two-way ANOVA with Bonferroni *post-hoc* tests and one-way ANOVA with the Newman–Keuls multiple comparison test.

In the single-dose toxicity analysis, the data showed no mortality and no drug-related toxicity signs in the tested mice at all tested doses within 14 days. Moreover, there were no significant differences between the groups in terms of body weight gain. Macroscopic examination revealed no differences in the physical appearance of the liver, heart, kidneys, spleen, lungs, and intestines between the treated groups and the control group (data not shown). In the repeat-dose toxicity analysis, the treated rats and the controls appeared uniformly healthy, and no lethality was recorded in the rats during the 28-day treatment period. There were no clinically abnormal symptoms in the general behavior between the treatment and control groups. In comparison with the control rats, the body weights of the female and male rats gradually increased during the test period. However, there was no statistically significant difference in body weight gain between the treated and control groups (**Figure 6A**). Similarly, no significant differences in the organ weights of rats (both males and females) were observed between the treated groups and the control group (**Figure 6B**). The indicators of the hematological and biochemical parameters of rats, which were not found to be significantly different between the vaccinated and control groups, are shown in **Figures 6C, D**, respectively. The macroscopic observations showed that there were no lesions or abnormalities in the physical appearance of the liver, kidneys, and spleen in any of the groups. As illustrated in **Figure 6E**, no significant macroscopic changes, such as color, size, shape, and texture, in the liver, spleen, and kidneys were observed between the groups. Similarly, the histopathological analysis results revealed that none of the kidney, liver, and spleen tissues of rats (both male and female) that received Nanocovax daily at all doses showed any damage or pathological alterations under microscopic observation (**Figure 6F**). These results indicate that both single- and repeat-dose toxicity studies in animals with all of the Nanocovax testing doses were safe.

## 4 Discussion

The development of vaccines with high immunogenicity and safety is a major concern for the control of the global COVID-19 pandemic and protection against further illness and fatalities. Vaccine candidates are currently under development using different platforms, such as inactivated vaccines, live-attenuated vaccines, viral vector (adenovirus) vaccines, DNA vaccines, and mRNA vaccines. In other platforms, recombinant protein vaccines were used together with adjuvants to enhance adaptive immunity. Aluminum salt-based adjuvants are approved for many human vaccines, such as those against hepatitis A, hepatitis B, *Haemophilus influenzae* type b (Hib), and diphtheria–tetanus–pertussis (DtaP, Tdap) (19). Currently, aluminum adjuvants have been applied in SARS-CoV-2 vaccines, such as RBD vaccines, S protein vaccines, inactivated virus vaccines, and virus-like particle (VLP)-based vaccines (20). For the Nanocovax vaccine, the S protein was designed (**Figure 1**) and first harvested at the final concentration (21.06 g/batch) after purification with 96.56% purity (**Figure 2**). Second, the S protein was formulated with an aluminum hydroxide adjuvant for preclinical research.

To evaluate the immunogenicity of the Nanocovax vaccine, three animal models (BALB/c mouse, Syrian hamster, and non-human primate) were utilized. The levels of S protein-specific IgG titer in the sera from vaccinated BALB/c mouse groups with various doses of Nanocovax (25, 50, 75, and 100 µg) significantly increased in a dose-dependent manner on day 14 post priming injection (**Figure 3A**). However, the levels of S protein-specific IgG titer were similar on days 28 and 45 in the hamster model (**Figure 3B**). In addition, the amounts of S protein-specific IgG in the sera of monkeys in all vaccinated groups were significant in a time-dependent manner (**Figure 3C**).

Neutralizing antibodies against SARS-CoV-2 are urgently needed to determine not only the infection rate and immunity but also the vaccine efficacy during clinical trials (21). Sera were collected from all vaccinated animal models to measure the percentage of neutralizing antibodies by a surrogate virus neutralization test. The results shown in **Figure 4** indicate that the antibodies in the sera can neutralize the SARS-CoV-2 virus with a high percentage of inhibition. In agreement with the results of Bošnjak et al. (9), a strong positive correlation between the surrogate virus neutralization test and the levels of S-specific IgG was observed. Our animal models indicated that IgG levels in the sera from animals vaccinated with Nanocovax showed increased high-affinity viral neutralizing antibodies. Furthermore, SARS-CoV-2-neutralizing antibodies can be measured using PRNT_50_. Here, sera from the BALB/c mice and hamster models had neutralizing antibody titers of 80–640 and 20–320, respectively (**Figure 4**).

The protective efficacy of the Nanocovax vaccine on hamsters was observed in this study. After challenge with SARS-CoV-2 virus, no symptoms, such as shortness of breath, ruffled fur, or lethargy, were observed. In addition, the weight loss was greater in the control hamster groups than in all vaccinated mice groups. On day 28, SARS-CoV-2 virus-specific RNA was detected in the lung samples of the non-vaccinated groups by real-time RT-PCR (**Figure 5**).

The safety of the Nanocovax vaccine was investigated based on single- and repeat-dose toxicity studies. The results showed that the Nanocovax vaccine has no single- or repeat-dose toxicity effects in mouse (*Mus musculus* var. albino) and rat (*Rattus norvegicus*) at four doses (25, 50, 75, and 100 µg).

Taken together, the Nanocovax vaccine demonstrated immunogenicity and safety based on animal models, including a non-human primate model. These results support the clinical phase I and phase II development of the Nanocovax vaccine.

## Data Availability Statement

The datasets presented in this study can be found in online repositories. The names of the repository/repositories and accession number(s) can be found in the article/**Supplementary Material**.

## Ethics Statement

The animal study was reviewed and approved by the ethics committee of Ministry of Health-Vietnam.

## Author Contributions

All authors contributed to the article and approved the submitted version.

## Funding

This study was supported by the Ministry of Science and Technology, Vietnam, under grant number: ĐTĐL.CN.51/20.

## Conflict of Interest

All authors except TTL, DCV and LKHN were employed by Nanogen Pharmaceutical Biotechnology Joint Stock Company (JSC).

The remaining authors declare that the research was conducted in the absence of any commercial or financial relationships that could be construed as a potential conflict of interest.

## Publisher’s Note

All claims expressed in this article are solely those of the authors and do not necessarily represent those of their affiliated organizations, or those of the publisher, the editors and the reviewers. Any product that may be evaluated in this article, or claim that may be made by its manufacturer, is not guaranteed or endorsed by the publisher.

## Abbreviations

COVID-19, coronavirus disease 2019; SARS-CoV-2, severe acute respiratory syndrome coronavirus 2; RBD, receptor-binding domain; ACE2, angiotensin-converting enzyme 2; CHO, Chinese hamster ovary.
