# Supplementary material for: Preclinical Immune Response and Safety Evaluation of the Protein Subunit Vaccine Nanocovax for COVID-19
Source: Front Immunol. 2021 Dec 6;12:766112. doi: 10.3389/fimmu.2021.766112 (PMC8685539; doi:10.3389/fimmu.2021.766112)
Supplement: Supplementary file 1 [file DataSheet_1.pdf]

Analysis report P0190-N006-02

Intact mass analysis of spike protein SARS-CoV-2 by MALDI-MS

Version 1

16.04.2021

**Customer**	**Service Provider**
Nanogen Pharmaceutical	Biofidus AG
Biotechnology JSC	Morgenbreede 1
Lot I – 5C Saigon Hitech Park	33615 Bielefeld
District 9	Germany
Ho Chi Minh City	
Vietnam	

Project manager: B. Müller 16.04.2021

Dr. Benjamin Müller Date

Content

Content 2

1 Executive summary 3

2 Aim 4

3 Abbreviations 4

4 Samples 4

5 Methods 4

5.1 Sample measurement with MALDI-MS 4

6 Results 5

6.1 Sample recombinant SARS-Cov-2-Spike Protein (PN006-2021-001) 5

1 Executive summary

Aim of the project was the intact mass analysis of a recombinant SARS-CoV-2 spike protein by MALDI-MS.

The MALDI-MS measurements were performed successfully for the sample. The average protein mass in the sample was determined to be **185668 ± 1849 Da**. The theoretical mass without posttranslational modifications according to the amino acid sequence is 136 kDa. The observed mass of 186 kDa suggests a high abundance of N-glycosylation which is within the expectations.

2 Aim

Aim of the project was the intact mass analysis of a recombinant SARS-CoV-2 spike protein by MALDI-MS.

3 Abbreviations

MALDI	Matrix assisted laser desorption ionization
MS	Mass spectrometry
TOF	Time of flight

4 Samples

The following sample was used for protein analysis.

Sample-ID Biofidus	Entry date	Sample ID customer	Description	Storage
PN006-2021-001	01.04.2021	recombinant SARS-Cov-2-Spike Protein	H.S. Code 38220000<br>Batch No. RSP2012004<br>Manuf.Date 29/12/2020	2-8°C

5 Methods

5.1 Sample measurement with MALDI-MS

The sample was desalted and concentrated with C4 ZipTips (Merck Millipore) and spotted on a ground steel target using 2',5'-Dihydroxyacetophenone. The sample was measured via MALDI-TOF-MS (UltrafleXtreme, Bruker Daltonik) in positive ion mode. Recorded MS spectra were processed by the software FlexAnalysis (Bruker Daltonik).

6 Results

6.1 Sample recombinant SARS-Cov-2-Spike Protein (PN006-2021-001)

The MALDI-MS measurement was performed successfully.

The exemplary smoothed mass spectrum is shown in Figure 1. The annotation of the mass peaks in the triplicate measurement and calculation of the average protein mass are shown in Table 1. The average protein mass in the sample was determined to be **185668 ± 1849 Da**.

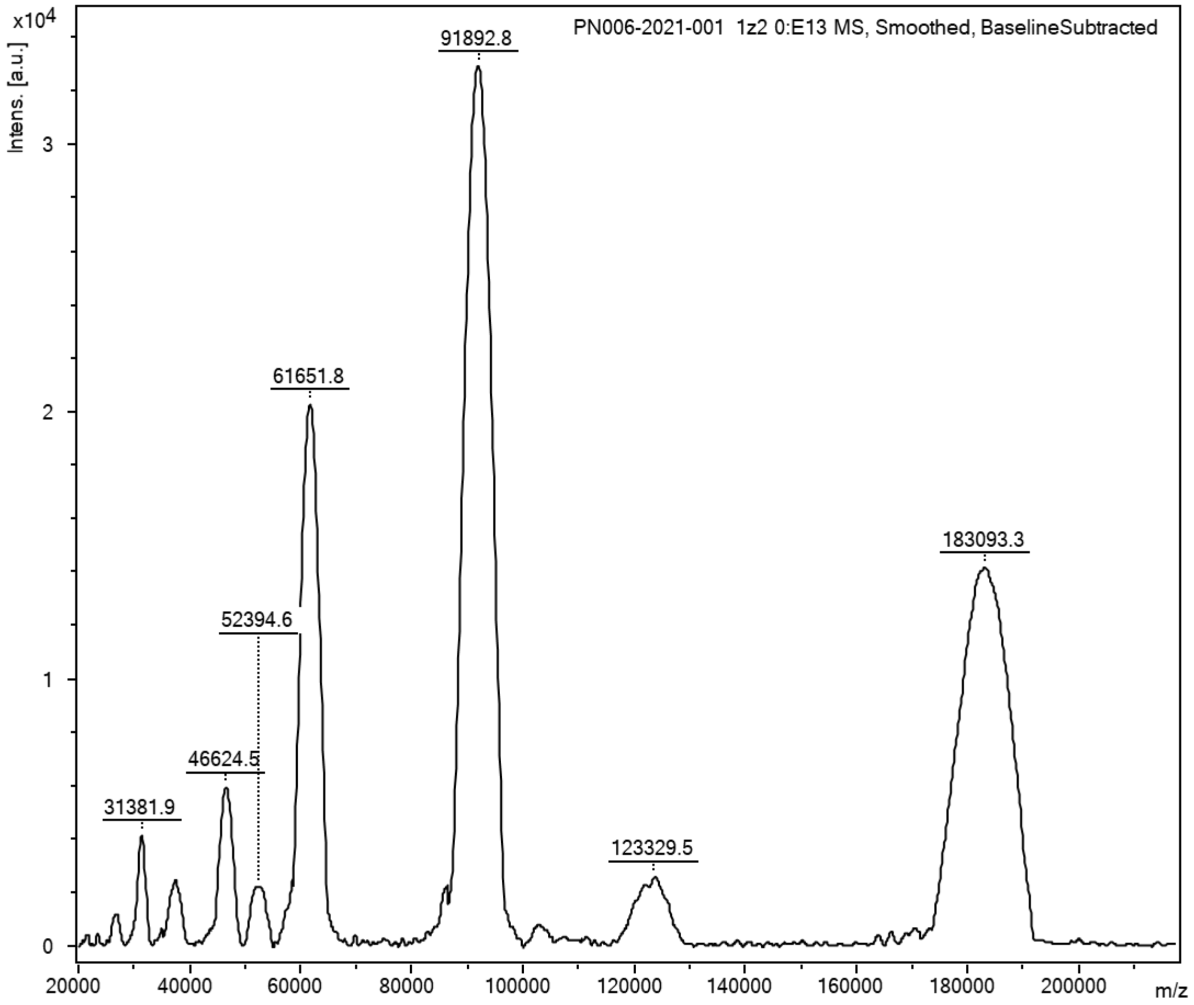

Figure 1: Exemplary smoothed mass spectrum of sample recombinant SARS-Cov-2-Spike Protein (PN006-2021-001). Dimer formation possible (e.g. the peak at 123329.5 m/z is the dimer of the triply charged protein at 61651.8 m/z).

Table 1: Mass annotation of MALDI-MS measurement of sample recombinant SARS-Cov-2-Spike Protein (PN006-2021-001).

Replicate measurement	m/z	z	Mass [Da]
1	31401.7	6	188404
1	37424.8	5	187119
1	46605.6	4	186418
1	61651.8	3	184952
1	92053.0	2	184104
1	183745.2	1	183744
2	31381.9	6	188285
2	37433.2	5	187161
2	46624.5	4	186494
2	61651.8	3	184952
2	91892.8	2	183784
2	183093.3	1	183092
3	31401.2	6	188401
3	37431.6	5	187153
3	46561.7	4	186243
3	61618.1	3	184851
3	91869.9	2	183738
3	183127.4	1	183126
**Average**	**185668 ± 1849 Da**		
