# Supplementary material for: Preclinical Immune Response and Safety Evaluation of the Protein Subunit Vaccine Nanocovax for COVID-19
Source: Front Immunol. 2021 Dec 6;12:766112. doi: 10.3389/fimmu.2021.766112 (PMC8685539; doi:10.3389/fimmu.2021.766112)
Supplement: Supplementary file 2 [file DataSheet_2.pdf]

Analysis report P0190-N006-01

Peptide mapping of spike protein SARS-CoV-2

by LC-ESI-MS

Version 1

16.04.2021

Customer

Nanogen Pharmaceutical
Biotechnology JSC
Lot I – 5C Saigon Hitech Park
District 9
Ho Chi Minh City
Vietnam

Service Provider

Biofidus AG
Morgenbreede 1
33615 Bielefeld
Germany

Project manager: B. Müller — 16.04.2021

Dr. Benjamin Müller — Date

Content

Content 2

1 Executive summary 3

2 Aim 4

3 Abbreviations 4

4 Samples 4

5 Methods 5

5.1 N-Deglycosylation 5

5.2 Reduction and alkylation 6

5.3 Enzymatic digestion 6

5.4 Sample measurement with LC-ESI-MS 6

5.5 Data analysis 6

6 Results 7

6.1 Sequence verification 7

6.2 N- and C-terminal modifications 9

7 Summary 11

8 Appendix 12

1 Executive summary

Aim of the project was the peptide mapping of a recombinant SARS-CoV-2 spike protein by LC-ESI-MS using different digestion strategies. Focus of the peptide mapping was:

- Sequence verification
- Analysis of N- and C-terminal modifications

The LC-ESI-MS measurements were performed successfully. The following observations were made:

- Aside from a truncated N-Terminus and a heterogeneous C-terminus, the **complete sequence was verified** for the SARS-CoV-2 spike protein.
- The mass spectrometric data suggests a **truncation of N-terminal serine** (compared to the theoretical N-terminus of the SARS-CoV-2 spike protein without the signal sequence as published for UnitProtKB entry P0DTC2) and complete **pyroglutamate formation of the N-terminal glutamine**.
- The **C-terminus** shows a high heterogeneity with a C-terminal peptide with truncation of AA 1215-1222 as the most abundant variant.

2 Aim

Aim of the project was the peptide mapping of a recombinant SARS-CoV-2 spike protein by LC-ESI-MS using different digestion strategies. Focus of the peptide mapping was:

- Sequence verification
- Analysis of N- and C-terminal modifications

3 Abbreviations

AA	Amino acid
DTT	Dithiothreitol
ESI	Electrospray ionization
FA	Formic acid
HPLC	High performance liquid chromatography
IAA	Iodoacetamide
LC	Liquid chromatography
MS	Mass spectrometry
MWCO	Molecular weight cut-off
QTOF	Quadrupol time of flight
TFA	Trifluoroacetic acid

4 Samples

The following sample was used for protein analysis.

Sample-ID Biofidus	Entry date	Sample ID customer	Description	Storage
PN006-2021-001	01.04.2021	recombinant SARS-Cov-2-Spike Protein	H.S. Code 38220000<br>Batch No. RSP2012004<br>Manuf.Date 29/12/2020	2-8°C

The following sequence was used for data analysis:

Spike protein SARS-CoV-2:

MFVFLVLLPL VSSQCVNLTT RTQLPPAYTN SFTRGVYYPD KVFRSSVLHS
TQDLFLPFFS NVTWFHAIHV SGTNGTKRFD NPVLPFNDGV YFASTEKSNI
IRGWIFGTTL DSKTQSLLIV NNATNVVIKV CEFQFCNDPF LGVYYHKNNK
SWMESEFRVY SSANNCTFEY VSQPFLMDLE GKQGNFKNLR EFVFKNIDGY
FKIYSKHTPI NLVRDLPQGF SALEPLVDLP IGINITRFQT LLALHRSYLT
PGDSSSGWTA GAAAYYVGYL QPRTFLLKYN ENGTITDAVD CALDPLSETK
CTLKSFTVEK GIYQTSNFRV QPTESIVRFP NITNLCPFGE VFNATRFASV
YAWNRKRISN CVADYSVLYN SASFSTFKCY GVSPTKLNDL CFTNVYADSF
VIRGDEVRQI APGQTGKIAD YNYKLPDDFT GCVIAWNSNN LDSKVGGNYN
YLYRLFRKSN LKPFERDIST EIYQAGSTPC NGVEGFNCYF PLQSYGFQPT
NGVGYQPYRV VVLSFELLHA PATVCGPKKS TNLVKNKCVN FNFNGLTGTG
VLTESNKKFL PFQQFGRDIA DTTDAVRDPQ TLEILDITPC SFGGVSVITP
GTNTSNQVAV LYQDVNCTEV PVAIHADQLT PTWRVYSTGS NVFQTRAGCL
IGAEHVNNSY ECDIPIGAGI CASYQTQTNS PRRARSVASQ SIIAYTMSLG
AENSVAYSNN SIAIPTNFTI SVTTEILPVS MTKTSVDCTM YICGDSTECS
NLLLQYGSFC TQLNRALTGI AVEQDKNTQE VFAQVKQIYK TPPIKDFGGF
NFSQILPDPS KPSKRSFIED LLFNKVTLAD AGFIKQYGDC LGDIAARDLI
CAQKFNGLTV LPPLLTDEMI AQYTSALLAG TITSGWTFGA GAALQIPFAM
QMAYRFNGIG VTQNVLYENQ KLIANQFNSA IGKIQDSLSS TASALGKLQD
VVNQNAQALN TLVKQLSSNF GAISSVLNDI LSRLDKVEAE VQIDRLITGR
LQSLQTYVTQ QLIRAAEIRA SANLAATKMS ECVLGQSKRV DFCGKGYHLM
SFPQSAPHGV VFLHVTYVPA QEKNFTTAPA ICHDGKAHFP REGVFVSNGT
HWFVTQRNFY EPQIITTDNT FVSGNCDVVI GIVNNTVYDP LQPELDSFKE
ELDKYFKNHT SPDVDLGDIS GINASVVNIQ KEIDRLNEVA KNLNESLIDL
QELGKYEQYI KWPRRRRRRR RR

5 Methods

5.1 N-Deglycosylation

The samples were N-deglycosylated with PNGase F at enzyme specific conditions.

5.2 Reduction and alkylation

The samples were reduced and denatured with DTT in the presence of urea or GuHCl. The samples were then alkylated with IAA.

5.3 Enzymatic digestion

The samples were enzymatically digested with the following enzymes at enzyme specific conditions to achieve a high sequence coverage:

- Trypsin
- Chymotrypsin
- AspN

5.4 Sample measurement with LC-ESI-MS

The samples were acidified in approx. 0.5% TFA and separated on a HPLC-system (Agilent 1100) using a reversed phase column (AdvanceBio Peptide Map 2.1 x 100 mm, 2.7 µm, Agilent). Eluents were 0.1% FA in water and 0.1% FA in acetonitrile. The mass spectrometric analysis was performed with a Compact QTOF mass spectrometer (Bruker Daltonik).

5.5 Data analysis

The recorded LC-ESI-MS and -MS/MS spectra were processed, annotated and searched against a customized sequence database using Mascot (Matrix Science). The following modifications were taken into consideration:

- Carbamidomethyl (C)
- Deamidation (NQ)
- Oxidation (M)
- N-terminal pyroglutamate formation (Q)

Modified peptides were identified by their exact mass and retention time and quantified by their mass spectrometric signal intensity.

6 Results

6.1 Sequence verification

The LC-ESI-MS measurements were performed successfully. An exemplary fragment mass spectrum of the peptide SFIEDLLFNKVTLADAGFIK (AA 816-835) is shown in Figure 1.

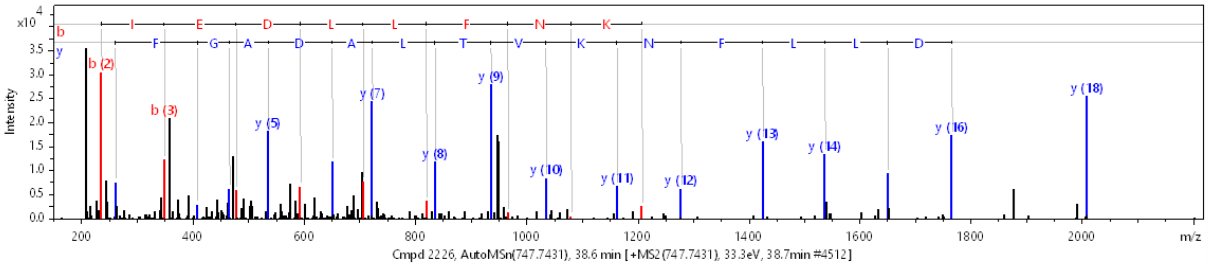

Figure 1: Exemplary fragment mass spectrum of peptide AGGVLVASHLQSFLEVSYR (AA 816-835) of sample recombinant SARS-Cov-2-Spike Protein (PN006-2021-001). Annotated are b- and y-ion series of the fragmentation pattern.

Aside from a truncated N-Terminus and a heterogeneous C-terminus, **complete sequence coverage** was achieved for the SARS-CoV-2 spike protein. The following modifications were observed:

- N-terminal pyroglutamate
- Several oxidations and deamidations

The full sequence data obtained with LC-ESI-MS measurements is shown in Figure 2. The sequence was matched to the sequence of the SARS-CoV-2 spike protein. The sequence region AA 708-718 (SNNSIAIPTNF) was verified manually (data not shown).

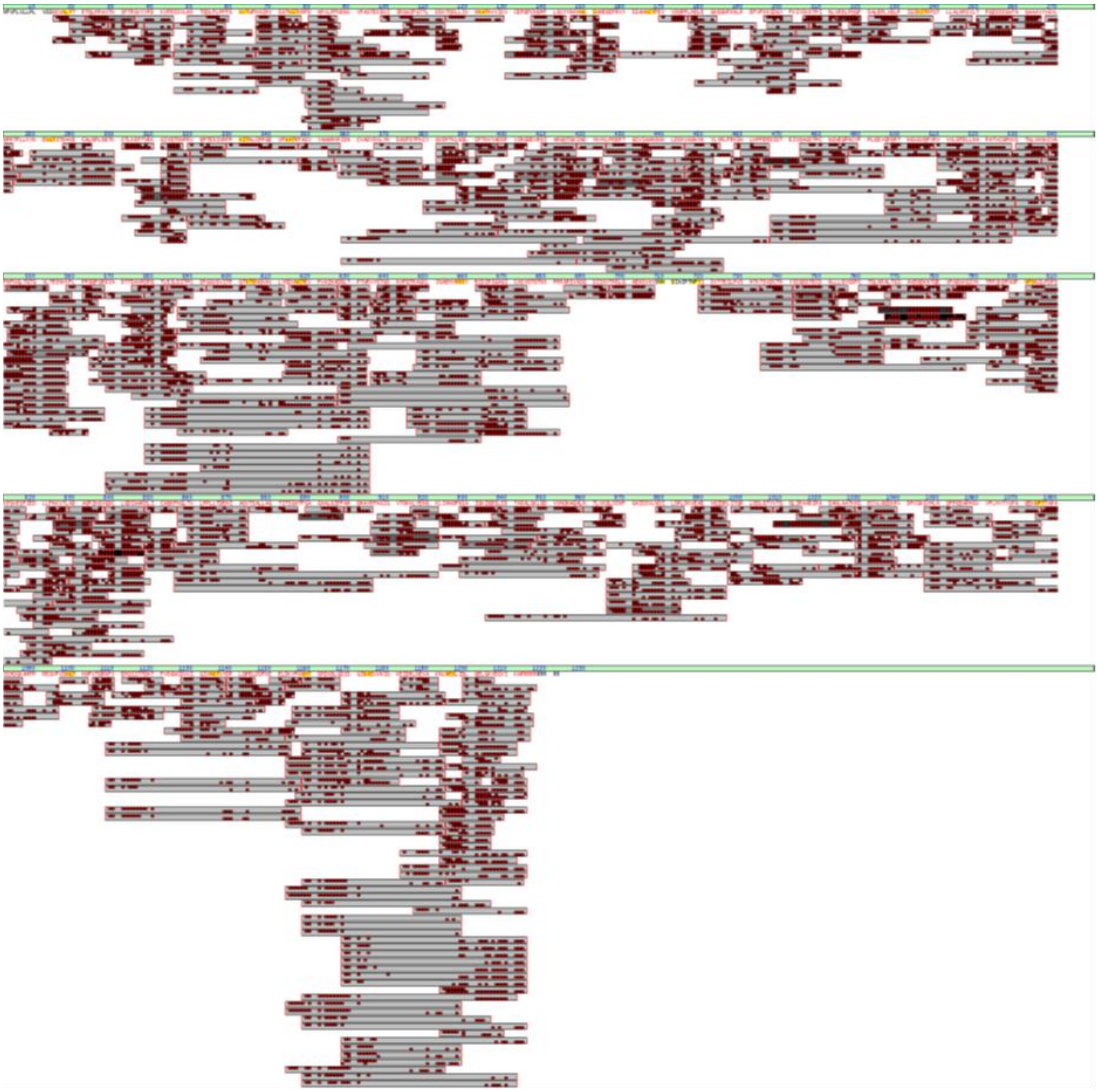

Figure 2: Results of amino acid sequencing of SARS-CoV-2 spike protein by LC-ESI-MS/MS for sample recombinant SARS-Cov-2-Spike Protein (PN006-2021-001). Grey boxes indicate proteolytic peptides analyzed with LC-MS. Red boxes within grey boxes indicate peptide fragments identified with LC-MS/MS.

6.2 N- and C-terminal modifications

The LC-ESI-MS measurements were performed successfully. An exemplary fragment mass spectrum of the C-terminal peptide DLQELGKYEQYIKWPR (AA 1199-1214) with truncation of 8 C-terminal arginines (AA 1215-1222) is shown in Figure 3. The exemplary extracted ion chromatograms of the C-terminal peptides are shown in Figure 4 for sample recombinant SARS-Cov-2-Spike Protein (PN006-2021-001). The mass spectrometric signal intensities suggest 51.5% C-terminal peptide with truncation of AA 1215-1222 in this sample. Additionally, further truncated C-terminal peptides have been observed.

For the N-terminus, the mass spectrometric data suggest a truncation of the N-terminal serine (compared to the theoretical N-terminus of the SARS-CoV-2 spike protein without the signal sequence as published for UnitProtKB entry P0DTC2) and N-terminal pyroglutamate formation. No further N-terminal modifications were observed.

The complete results of the analysis of N- and C-terminal modifications for the sample are summarized in Table 1. The peptide masses used for analysis of N- and C-terminal modifications are summarized in Table 2 in the appendix.

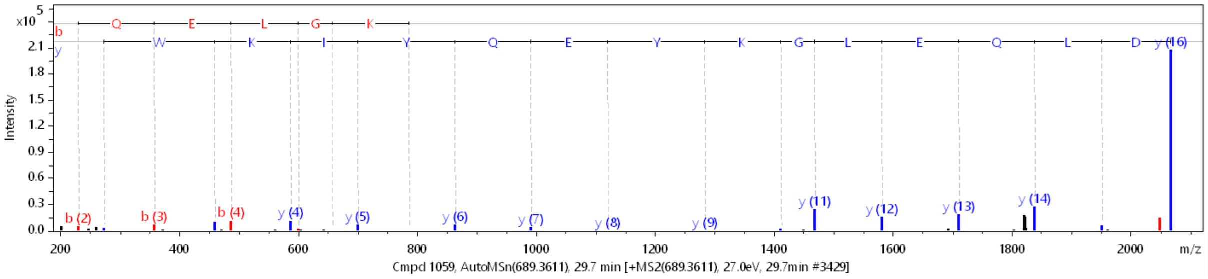

Figure 3: Exemplary fragment mass spectrum of the C-terminal peptide DLQELGKYEQYIKWPR (AA 1199-1214) of sample recombinant SARS-Cov-2-Spike Protein (PN006-2021-001) with truncation of 8 C-terminal arginines (AA 1215-1222). Annotated are b- and y-ion series of the fragmentation pattern.

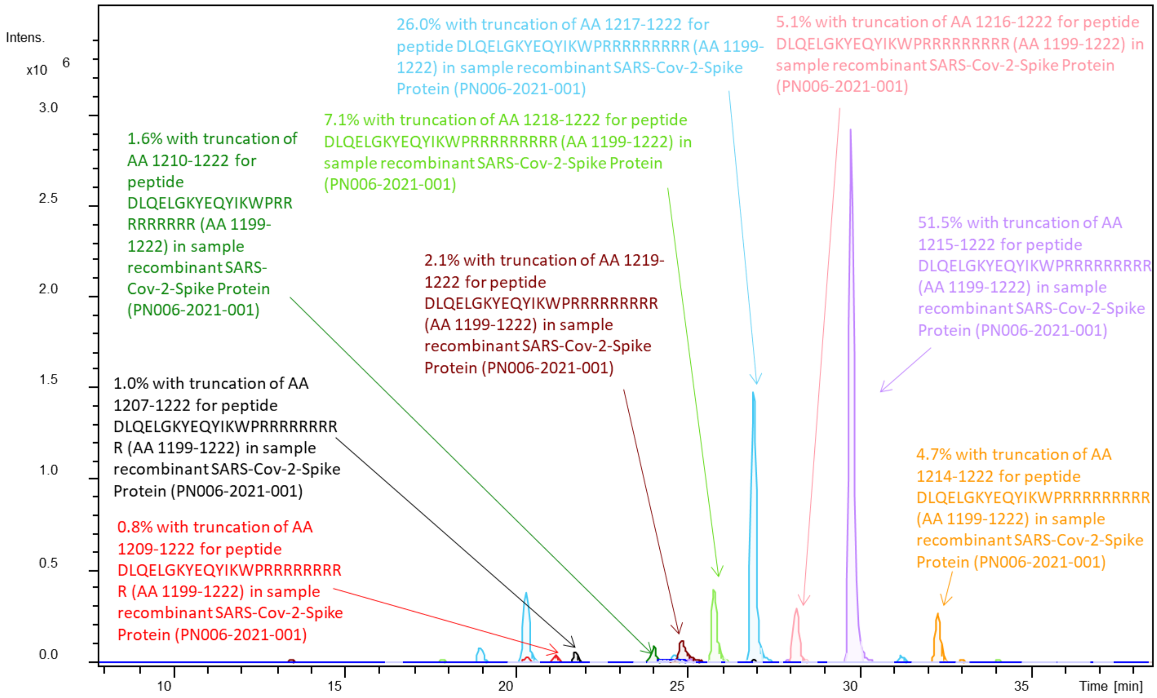

Figure 4: Exemplary extracted ion chromatograms of C-terminal peptide DLQELGKYEQYIKWPRRRRRRRRR (AA 1199-1222) with truncation of AA 1207-1222 (black), truncation of AA 1209-1222 (red), truncation of AA 1210-1222 (dark green), truncation of AA 1214-1222 (orange), truncation of AA 1215-1222 (violet), truncation of AA 1216-1222 (pink), truncation of AA 1217-1222 (cyan), truncation of AA 1218-1222 (light green) and truncation of AA 1219-1222 (brown) of sample recombinant SARS-Cov-2-Spike Protein (PN006-2021-001). Based on mass spectrometric signal intensity the level of truncation of AA 1207-1222 was observed at 1.0%, truncation of AA 1209-1222 at 0.8%, truncation of AA 1210-1222 at 1.6%, truncation of AA 1214-1222 at 4.7%, truncation of AA 1215-1222 at 51.5%, truncation of AA 1216-1222 at 5.1%, truncation of AA 1217-1222 at 26.0%, truncation of AA 1218-1222 at 7.1% and truncation of AA 1219-1222 at 2.1%.

Table 1: Summary of analysis of N- and C-terminal heterogeneity by LC-ESI-MS for the sample recombinant SARS-Cov-2-Spike Protein (PN006-2021-001).

Terminus	Peptide	Range	Modification	Relative intensity based on MS signal
N-terminus	<u>Q</u>CVNLTTR (=pECVNLTTR)	14-21	N-terminal pyroglutamate and truncation of AA 1-13	100.0%
C-terminus	DLQELGKYEQYIKWPRRRRR	1199-1218	Truncation of AA 1219-1222	2.1%
	DLQELGKYEQYIKWPRRRR	1199-1217	Truncation of AA 1218-1222	7.1%
	DLQELGKYEQYIKWPRRR	1199-1216	Truncation of AA 1217-1222	26.0%
	DLQELGKYEQYIKWPRR	1199-1215	Truncation of AA 1216-1222	5.1%
	DLQELGKYEQYIKWPR	1199-1214	Truncation of AA 1215-1222	51.5%
	DLQELGKYEQYIKWP	1199-1213	Truncation of AA 1214-1222	4.7%
	DLQELGKYEQY	1199-1209	Truncation of AA 1210-1222	1.6%
	DLQELGKYEQ	1199-1208	Truncation of AA 1209-1222	0.8%
	DLQELGKY	1199-1206	Truncation of AA 1207-1222	1.0%

7 Summary

Aim of the project was the peptide mapping of a recombinant SARS-CoV-2 spike protein by LC-ESI-MS using different digestion strategies. Focus of the peptide mapping was:

- Sequence verification
- Analysis of N- and C-terminal modifications

The LC-ESI-MS measurements were performed successfully. The following observations were made:

- Aside from a truncated N-Terminus and a heterogeneous C-terminus, the **complete sequence was verified** for the SARS-CoV-2 spike protein.
- The mass spectrometric data suggests a **truncation of N-terminal serine** (compared to the theoretical N-terminus of the SARS-CoV-2 spike protein without the signal sequence as published for UnitProtKB entry P0DTC2) and complete **pyroglutamate formation of the N-terminal glutamine**.
- The **C-terminus** shows a high heterogeneity with a C-terminal peptide with truncation of AA 1215-1222 as the most abundant variant.

8 Appendix

Table 2: Masses of analyzed peptides of N- and C-terminus by LC-ESI-MS for the sample recombinant SARS-Cov-2-Spike Protein (PN006-2021-001).

Terminus	Peptide	Range	Theoretical mass [Da]
N-terminus	QCVNLTTR (=pECVNLTTR)	14-21	974.4491
C-terminus	DLQELGKYEQYIKWPRRRRR	1199-1218	2689.4677
	DLQELGKYEQYIKWPRRRR	1199-1217	2533.3666
	DLQELGKYEQYIKWPRRR	1199-1216	2377.2655
	DLQELGKYEQYIKWPRR	1199-1215	2221.1644
	DLQELGKYEQYIKWPR	1199-1214	2065.0633
	DLQELGKYEQYIKWP	1199-1213	1908.9622
	DLQELGKYEQY	1199-1209	1384.6511
	DLQELGKYEQ	1199-1208	1221.5877
	DLQELGKY	1199-1206	964.4866

Table 3: Attached files.

Description	File
Mascot results	RAW_P0190-N006-01 Sequence Verification Mascot Results PN006-2021-001_001_210416_BMu.pdf
